# *Plasmodium* 18S Ribosomal RNA Biomarker Clearance After Food and Drug Administration–Approved Antimalarial Treatment in Controlled Human Malaria Infection Trials

**DOI:** 10.1093/ofid/ofad202

**Published:** 2023-04-13

**Authors:** Chris Chavtur, Weston J Staubus, Mabel Ho, Dianna E B Hergott, Annette M Seilie, Sara Healy, Patrick Duffy, Lisa Jackson, Angela Talley, Stefan H I Kappe, Stephen L Hoffman, Thomas L Richie, James G Kublin, Ming Chang, Sean C Murphy

**Affiliations:** Malaria Molecular Diagnostic Laboratory, Department of Laboratory Medicine and Pathology and Center for Emerging and Re-emerging Infectious Diseases, University of Washington, Seattle, Washington, USA; Malaria Molecular Diagnostic Laboratory, Department of Laboratory Medicine and Pathology and Center for Emerging and Re-emerging Infectious Diseases, University of Washington, Seattle, Washington, USA; Malaria Molecular Diagnostic Laboratory, Department of Laboratory Medicine and Pathology and Center for Emerging and Re-emerging Infectious Diseases, University of Washington, Seattle, Washington, USA; Malaria Molecular Diagnostic Laboratory, Department of Laboratory Medicine and Pathology and Center for Emerging and Re-emerging Infectious Diseases, University of Washington, Seattle, Washington, USA; Department of Epidemiology, School of Public Health, University of Washington, Seattle, Washington, USA; Malaria Molecular Diagnostic Laboratory, Department of Laboratory Medicine and Pathology and Center for Emerging and Re-emerging Infectious Diseases, University of Washington, Seattle, Washington, USA; Laboratory of Malaria Immunology and Vaccinology, National Institute of Allergy and Infectious Diseases, Bethesda, Maryland, USA; Laboratory of Malaria Immunology and Vaccinology, National Institute of Allergy and Infectious Diseases, Bethesda, Maryland, USA; Kaiser Permanente Washington Health Research Institute, Seattle, Washington, USA; Spero Therapeutics, Cambridge, Massachusetts, USA; Center for Global Infectious Disease Research, Seattle Children's Research Institute, Seattle, Washington, USA; Department of Pediatrics, University of Washington, Seattle, Washington, USA; Sanaria, Rockville, Maryland, United States; Sanaria, Rockville, Maryland, United States; Vaccine and Infectious Disease Division, Fred Hutchinson Cancer Research Center, Seattle, Washington, USA; Seattle Malaria Clinical Trials Center, Fred Hutchinson Cancer Research Center, Seattle, Washington, USA; Malaria Molecular Diagnostic Laboratory, Department of Laboratory Medicine and Pathology and Center for Emerging and Re-emerging Infectious Diseases, University of Washington, Seattle, Washington, USA; Malaria Molecular Diagnostic Laboratory, Department of Laboratory Medicine and Pathology and Center for Emerging and Re-emerging Infectious Diseases, University of Washington, Seattle, Washington, USA; Seattle Malaria Clinical Trials Center, Fred Hutchinson Cancer Research Center, Seattle, Washington, USA; Department of Microbiology, University of Washington, Seattle, Washington, USA; Department of Laboratories, Seattle Children's Hospital, Seattle, Washington, USA

**Keywords:** 18S ribosomal RNA, controlled human malaria infection, biomarker clearance, RT-PCR, *Plasmodium*‌

## Abstract

**Background:**

Sensitive molecular assays, such as quantitative reverse-transcription polymerase chain reaction (qRT-PCR) of *Plasmodium* 18S ribosomal RNA (rRNA), are increasingly the primary method of detecting infections in controlled human malaria infection (CHMI) trials. However, thick blood smears (TBSs) remain the main method for confirming clearance of parasites after curative treatment, in part owing to uncertainty regarding biomarker clearance rates.

**Methods:**

For this analysis, 18S rRNA qRT-PCR data were compiled from 127 *Plasmodium falciparum*–infected participants treated with chloroquine or atovaquone-proguanil in 6 CHMI studies conducted in Seattle, Washington, over the past decade. A survival analysis approach was used to compare biomarker and TBS clearance times among studies. The effect of the parasite density at which treatment was initiated on clearance time was estimated using linear regression.

**Results:**

The median time to biomarker clearance was 3 days (interquartile range, 3–5 days), while the median time to TBS clearance was 1 day (1–2 days). Time to biomarker clearance increased with the parasite density at which treatment was initiated. Parasite density did not have a significant effect on TBS clearance.

**Conclusions:**

The *Plasmodium* 18S rRNA biomarker clears quickly and can be relied on to confirm the adequacy of Food and Drug Administration–approved treatments in CHMI studies at nonendemic sites.

## Teaser

Posttreatment *Plasmodium* 18S ribosomal RNA molecular diagnostic data were assessed from multiple controlled human malaria infection studies to ascertain the clearance kinetics of this increasingly used biomarker. All participants showed complete clearance by 7 days after treatment initiation.

The World Health Organization estimates there were 241 million cases of malaria in 2020, resulting in 627 000 deaths [1]. Better methods of control and prevention are urgently needed to reduce the global burden of this disease. A critical tool in the effort to develop such methods is the controlled human malaria infection (CHMI) trial. CHMI trials are studies that involve deliberate, controlled infection of human participants with *Plasmodium* spp. parasites under medical supervision to assess the in vivo efficacy of experimental drugs and vaccines [2]. They provide rapid, actionable results to prioritize candidates for later field studies.

Daily monitoring of participants after sporozoite challenge is standard practice in CHMI trials to diagnose infections and ensure that they clear following rescue treatment with approved antimalarial medications. For decades, microscopic examination of thick blood smears (TBSs) has been the reference standard method for these purposes owing to its simplicity, low cost, and efficacy for detecting infections associated with clinical illness [3]. In recent years, however, molecular assays, such as quantitative reverse-transcription polymerase chain reaction (qRT-PCR) targeting *Plasmodium* 18S ribosomal RNA (rRNA), have emerged as viable alternatives. Molecular assays offer several advantages over TBSs in the context of CHMI, including more precise quantification, less operator dependency, and greater sensitivity: whereas the limit of detection (LoD) for TBSs is parasite density of approximately 0.5–1 × 10^4^/mL of blood [4, 5], qRT-PCR can detect *Plasmodium* 18S rRNA in blood containing as few parasites as 10/mL [6]. This allows infections to be detected and treated earlier and can help identify participants with persistent, subpatent parasitemia [7, 8].

Based on extensive analytical and clinical data, the US Food and Drug Administration (FDA) qualified the *Plasmodium* 18S rRNA biomarker for diagnosis of *Plasmodium falciparum* infections in CHMI trial participants [7], and qRT-PCR or qPCR of the biomarker has subsequently replaced TBS as the primary tool for this purpose in several centers [8–13]. However, TBSs remain the primary means of confirming infection clearance following rescue treatment, in part because they detect whole parasites and are therefore highly specific for active infections. In contrast, molecular assays detect parasite components, which may remain detectable even after TBSs have cleared [14, 15]. Prolonged biomarker clearance is also well documented for antigen-based rapid diagnostic tests [16].

Here, data from CHMI trials conducted in the United States are used to investigate the persistence of *Plasmodium* 18S rRNA following treatment with chloroquine or atovaquone-proguanil and to assess its reliability as an indicator of infection clearance in the context of CHMI studies at nonendemic sites. The effect of parasite density on clearance is also explored.

## METHODS

### CHMI Trials

Data were compiled from 6 institutional review board–approved CHMI studies that were conducted in Seattle, Washington, from 2010 to 2019 and used *Plasmodium* 18S rRNA qRT-PCR as an end-point assay (Table 1) [8, 10, 11, 17–20]. Informed consent for each clinical study was described in previously published reports [8, 10, 11, 17–20]. The drug or vaccine administered to treatment groups, the time between administration and sporozoite challenge, and the mode of challenge (mosquito bite or direct venous injection) varied, but all 6 studies enrolled healthy, malaria-naive adults between 18 and 50 years old and used *P falciparum* NF54 for CHMI. Many of the participants who became infected were in infectivity control cohorts, but others received experimental vaccines or drugs before challenge.

**Table 1.** Summary of Controlled Human Malaria Infection Studies

Clinicaltrials.gov Identifier	Study Name	Rescue Treatment Criteria	Drug	Reference(s)
NCT01058226	MC-001/Demo	Positive TBS	Chloroquine	[17, 18]
NCT01500980	MC-003/Cvac	Positive TBS	Atovaquone-proguanil	[19]
NCT04072302	KAF156	2 consecutive positive qRT-PCR results with temperature <38.0°C, including 1 estimated parasite density ≥250/mL, *or* 1 positive qRT-PCR result with temperature ≥38.0°C *or* Positive TBS	Atovaquone-proguanil	[10]
NCT02562872	DSM265	1 positive qRT-PCR with estimated parasite density ≥250/mL	Atovaquone-proguanil	[8]
NCT02773979	VTEU 11-0042/Cvac	Groups 1 and 2: 2 positive qRT-PCR results within 60 h, including 1 estimated parasite density ≥250/mL; Group 3: 1 positive qRT-PCR result with estimated parasite density ≥20/mL	Atovaquone-proguanil	[20]
NCT03168854	VTEU 14-0088/PfGAP3KO	1 positive qRT-PCR result with estimated parasite density ≥20/mL *or* Positive TBS	Atovaquone-proguanil	[11]

Abbreviations: qRT-PCR, quantitative reverse-transcription polymerase chain reaction; TBS, thick blood smear.

Criteria for rescue treatment differed among the studies (Table 1). In the 2 earliest studies (which were intended to demonstrate the safety and feasibility of monitoring CHMI participants by qRT-PCR of *Plasmodium* 18S rRNA), treatment was initiated after a positive TBS (“TBS-defined studies”). In the 4 later studies, treatment was initiated after a participant's qRT-PCR–estimated parasite density crossed a predefined threshold (“biomarker-defined studies”). The highest threshold used in any of the biomarker-defined studies was 2 consecutive positive results, including 1 with an estimated parasite density ≥250/mL blood (still well below the TBS LoD), while the lowest was a single positive qRT-PCR result with an estimated parasite density ≥20/mL. Participants who reached the threshold were treated within 24 hours with a standard curative regimen of atovaquone-proguanil or, in the case of MC-001, chloroquine.

Participants were monitored after treatment to confirm that infections cleared. In the TBS-defined studies, blood samples were collected once or twice daily for immediate analysis by TBS. Blood sampling continued until parasites were not detected for 3 consecutive days. Analysis by qRT-PCR was performed post hoc, so the results did not inform the collection schedule. In the biomarker-defined studies, blood sampling was often designed to occur less frequently than every day. In addition, blood samples were analyzed by same-day qRT-PCR, and biomarker levels were used to guide further testing. As a result, participants sometimes went ≥2 days without testing between their last positive qRT-PCR result and subsequent negative result (the result marking clearance), leading to the likely overestimation of their biomarker clearance times. The number of participants with gaps of >1 day between those tests and the average gap are reported for each study. All participants in all studies were regularly monitored for malaria symptoms and tested by qRT-PCR at the end of the study (28–42 days after challenge) to ensure clearance of their infections.

### Patient Consent Statement

The original design of all included studies and consents for future research were approved by local ethical committees. Informed consent for each clinical study was described in previously published reports [8, 10, 11, 17–20]. No additional documentation was required to make the analyses reported herein.

### Statistical Analysis

Survival analysis (time-to-event analysis) was used to assess and compare TBS and biomarker clearance times. Participants were censored after their last positive test result if it was followed by a break in testing of ≥7 days. Median clearance times were calculated from Kaplan-Meier curves. Log-rank tests were used to detect differences between groups (α = .05). Pearson's correlation coefficients were calculated for the TBS and biomarker clearance times of uncensored participants in the TBS-defined studies. Unadjusted linear regression was performed to determine the effect of qRT-PCR–estimated, log_10_-transformed parasite density when treatment was initiated on the time to TBS and biomarker clearance. Only participants with quantifiable parasitemia at the time treatment was initiated and uncensored clearance data (ie, an estimate of TBS or biomarker clearance time known to be accurate to within 7 days) were included in the regression analyses. All analyses were performed using R Statistical Software, version 4.1.3 [21]. Survival analysis was conducted using the survival R package, version 3.2.13 [22].

## RESULTS

Across the 6 CHMI studies, 127 participants experienced *P falciparum* infections after challenge and received curative drug treatment (Table 2). One participant was excluded from all analyses because of persistent gametocytemia that required a second round of treatment [8, 23]. Of the 126 remaining participants, 59 (47%) were confirmed to have cleared the biomarker within 1 day of their last positive qRT-PCR test. Thirteen were not tested again for ≥7 days after their last positive qRT-PCR result and were censored. There were no notable differences between censored and uncensored participants with respect to sex, age, parasite density when treatment was initiated, or study arm (infectivity control or drug/vaccine). Four (25%), 6 (33%), and 44 (98%) participants in 14-0088, DSM265, and KAF156, respectively, who experienced shorter (1–3-day) gaps in testing immediately preceding the day the biomarker was confirmed cleared were not censored.

**Table 2.** *Plasmodium* Infection Treatment and Clearance Characteristics

Study Name	No. Treated for Infection (Diagnostic)	No. (%) Censored	Interval Between Last Positive qRT-PCR Result and Confirmation of Clearance, Mean (SD), d^a^	Time to TBS Clearance, Median (IQR), d	Time to Biomarker Clearance, Median (IQR), d
MC-001/Demo	6 (TBS)	3 (50)	1.0 (0)	2 (1–2)	4 (3 to NA)
MC-003/CVac	25 (TBS)	8 (32)	0.5 (0)	1 (1–2)	4 (3–4)
KAF156	47 (Biomarker)	2 (4)	2.9 (0.6)	…	3 (3–5)
DSM265	18^b^ (Biomarker)	0 (0)	1.5 (0.9)	…	3 (3–5)
VTEU 11-0042/CVac	15 (Biomarker)	0 (0)	1.0 (0)	…	3 (2–4)
VTEU 14-0088/PfGAP3KO	16 (Biomarker)	0 (0)	1.3 (0.6)	…	1.5 (1–4)
Total	127	13 (10)	1.8 (1.0)	1 (1–2)	3 (3–5)

Abbreviations: IQR, interquartile range; NA, not applicable (cannot be estimated owing to censoring); SD, standard deviation; TBS, thick blood smear.

a Censored participants are excluded.

b One of these participants was excluded from all analyses after development of persistent gametocytemia.

### TBS and 18S rRNA Biomarker Clearance After TBS-Initiated Treatment

**Figure 1.**
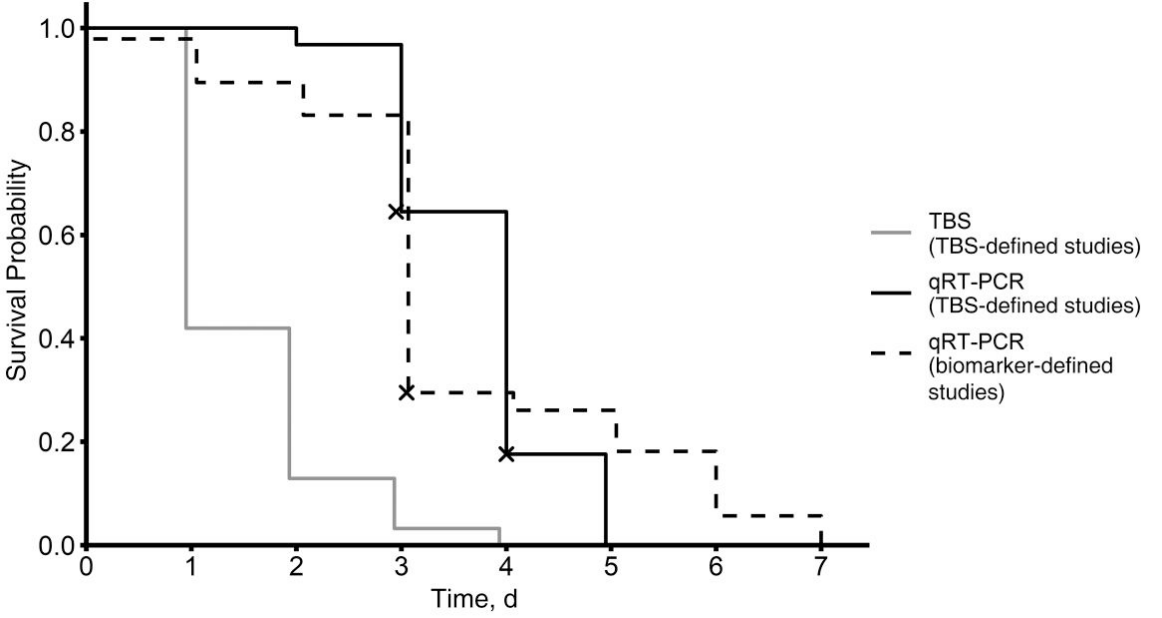
Kaplan-Meier plot showing the probability of being positive by thick blood smear (TBS) and 18S ribosomal RNA quantitative reverse-transcription polymerase chain reaction (qRT-PCR) for participants in TBS-defined and biomarker-defined studies. Each *X* indicates that ≥1 participant was censored at that time point.

All participants in the TBS-defined studies (n = 31) were TBS and biomarker positive when treatment was initiated. All remained biomarker positive for at least as many days as they remained TBS positive. A log-rank test showed that time to biomarker clearance was significantly longer than time to TBS clearance among these participants (χ^2^ = 47.3; *P* < .001). However, the difference was small relative to the duration of a CHMI trial: Kaplan-Meier curves (Figure 1) showed that the median time to TBS clearance was 1 day (interquartile range [IQR], 1–2 days), compared with 4 days (3–4 days) for biomarker clearance. Time to biomarker clearance was significantly correlated with time to TBS clearance (Pearson's *r* = 0.50; 95% confidence interval [CI], .07–.77).

### 18S rRNA Biomarker Clearance After qRT-PCR­–Initiated Treatment

Only biomarker clearance could be assessed for participants in the biomarker-defined studies (n = 95), as the high sensitivity of qRT-PCR and low treatment thresholds resulted in participants being treated before they became TBS positive. Kaplan-Meier curves showed that the median time to biomarker clearance among participants in the biomarker-defined studies was 3 days (IQR, 3–5 days), 1 day less than the median time to biomarker clearance among participants in the TBS-defined studies. Nevertheless, log-rank tests indicated that the time to biomarker clearance among participants in the biomarker-defined studies did not differ significantly from the time to biomarker clearance among participants in the TBS-defined studies (χ^2^ = 2.2; *P* = .14) and was significantly longer than the time to TBS clearance among participants in the TBS-defined studies (χ^2^ = 55.2; *P* < .001). The median times to TBS (if applicable) and biomarker clearance are listed along with the ranges and IQRs for each individual study and for participants overall in Table 2. Kaplan-Meier plots for each study are shown in Supplementary Figure 1.

### Effect of Parasite Density on Clearance

The parasite density at which treatment was initiated—as estimated by qRT-PCR—varied widely among study participants. In the TBS-defined studies, parasite densities ranged from 2945/mL to 321 345/mL. In the biomarker-defined studies, there were 4 participants (3 in 14-0088 and 1 in 11-0042) in cohorts for which the treatment threshold was a single positive qRT-PCR result with parasite density ≥20/mL who had unquantifiable parasitemia (<20/mL) on the day treatment was initiated (1 day after they met the treatment threshold). Among the rest, the parasite density at which treatment was initiated ranged from 36/mL to 177 341/mL.

**Figure 2.**
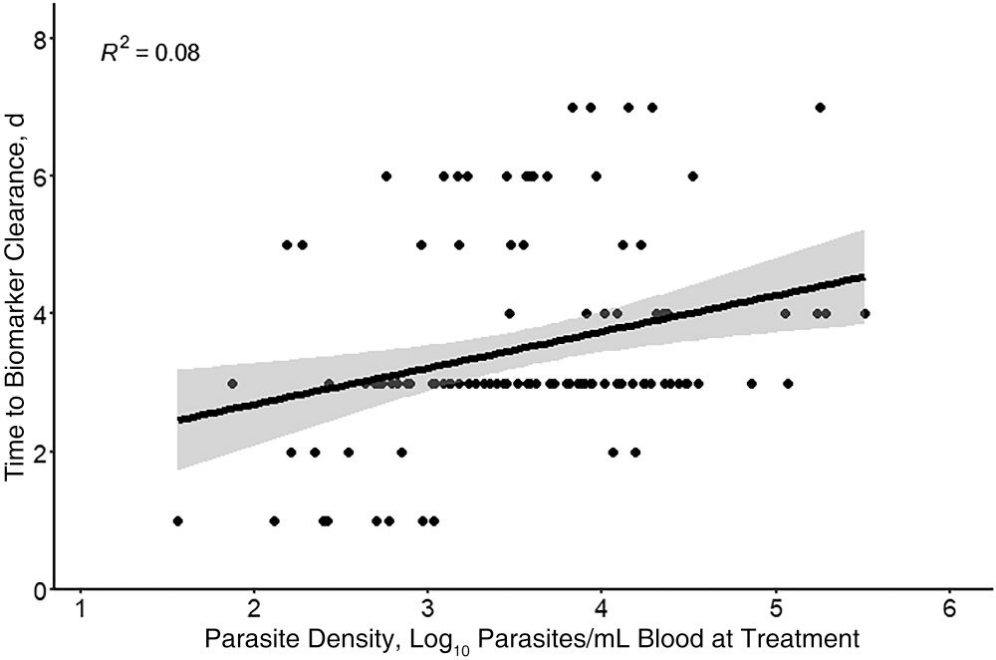
Time to biomarker clearance versus log_10­_-transformed parasite density at the time of treatment for participants in thick blood smear–based and 18S ribosomal RNA (rRNA)–based studies with complete 18S rRNA data and quantifiable parasitemia on the day of treatment. Line represents linear regression fit; shading, 95% confidence intervals.

Across all participants in all studies (TBS and biomarker defined) who had uncensored biomarker clearance data and quantifiable parasitemia when treatment was initiated (n = 109), linear regression showed that log_10_-transformed parasite density at the time treatment was initiated was a significant predictor of time to biomarker clearance (*P* = .006; Figure 2). For every 10-fold increase in parasite density, the biomarker clearance time was predicted to increase by an additional 0.52 days (95% CI, .15–.88 days). In contrast, for participants with TBS clearance data (TBS-defined studies only; n = 31), a 10-fold increase in parasite density was predicted to increase TBS clearance time by 0.09 days (95% CI, −.52 to .71 days), a difference that was not statistically significant (*P* = .76).

## DISCUSSION

Molecular assays are highly sensitive and widely used methods of diagnosing infection in malaria clinical trials. However, since some biomarkers can persist after successful treatment, it is important to understand their posttreatment dynamics before using them as a test of cure. In the current study, the rate of clearance of *P falciparum* 18S rRNA, a biomarker recently qualified by the FDA for detecting infections in CHMI trials at malaria-nonendemic sites, was evaluated using data from 6 CHMI studies conducted in Seattle in which qRT-PCR was used to monitor clearance of patent and subpatent infections. Not surprisingly, the biomarker remained detectable by highly sensitive qRT-PCR longer than parasites were detectable by TBS. However, the difference was only a few days, which could be explained in part by the additional time required for parasitemia to decrease from the LoD of TBSs to that of 18S rRNA qRT-PCR. The biomarker was not detected >1 week after treatment in any participant whose treatment was successful. Treatment in these studies was with either chloroquine or atovaquone-proguanil, though the findings are likely also applicable to other drugs such as artemether-lumefantrine. These findings support the conclusion that qRT-PCR of the 18S rRNA biomarker is a suitable method for monitoring posttreatment clearance.

Infections with higher initial densities are expected to take longer to clear [24]. Hence, it was surprising that while there was a significant relationship overall between parasite density when treatment was initiated and biomarker clearance time, survival analysis did not indicate a significant difference in biomarker clearance time between the studies that used TBSs to define infection and those that used qRT-PCR, given that the latter method enables earlier detection of infection [7]. One reason for this may have been that some of the biomarker-defined studies—including the largest of the 4 studies, KAF156—used relatively conservative treatment thresholds (2 consecutive positive results, including 1 with an estimated parasite density of ≥250/mL), which sometimes resulted in parasite densities near or above the LoD of TBSs by the time treatment was initiated.

Another reason is that intervals between blood sampling were often longer in the biomarker-defined studies than in the TBS-defined studies. For example, while participants in TBS-defined studies were tested for ≥3 consecutive days after treatment was initiated, participants in KAF156 were generally not tested until the third day after treatment was initiated, by which time many had already cleared the biomarker (Supplementary Figure 1). Moreover, investigators—armed with several days of biomarker data—tended to use greater discretion regarding additional testing as the number of days since treatment was initiated increased, leading to longer and more frequent gaps in testing. This likely led to overestimation of biomarker clearance times, as clearance of the biomarker might not have been confirmed until 1–3 days after the fact, and it may explain why the rate of biomarker clearance appears higher in biomarker-defined studies than in TBS-defined studies over the first 3 days but lower thereafter.

Overall, these data support the use of 18S rRNA qRT-PCR to monitor infections throughout CHMI studies at nonendemic sites. It has previously been shown that using the biomarker to monitor participants after challenge and diagnose infections leads to earlier treatment, lower maximum parasitemia, and fewer adverse effects [7]. The present findings suggest that it can also safely be used to follow up participants to clearance after end-of-study treatment with FDA-approved drugs. Furthermore, because qRT-PCR is more sensitive than TBS microscopy and quantitative across a wide range of parasite densities, biomarker-based monitoring protocols provide additional information about infections that could allow for reduced testing frequencies after treatment in CHMI studies. For example, since the biomarker appears to clear within 7 days under normal circumstances, testing could be scheduled for the last day of treatment to confirm declining parasitemia and 7 days after starting treatment to confirm clearance, with plans for additional testing in response to unexpected results (ie, rising parasitemia through treatment or incomplete clearance within 7 days), for new or persistent symptoms, or at the discretion of investigators. As shown in Table 3, CHMI trials conducted in Seattle over the past decade have already transitioned from TBS-based designs and daily posttreatment testing to qRT-PCR–based designs and limited posttreatment testing, saving time and resources for clinical trial sites and reducing the number of participant visits required.

**Table 3.** Changes in Posttreatment Monitoring Approach in Controlled Human Malaria Infection Studies at Nonendemic Sites Over Time

Study Name	Year(s)	Diagnostic Method(s)	Approach
MC-001/Demo	2010–2011	TBS	TBS daily until 3 d (consecutive) of negative results and all symptoms resolved
MC-003/Cvac	2012	TBS	TBS twice daily until 3 d (consecutive) of negative results and all symptoms resolved
KAF156	2014–2017	Primary: TBS; secondary: qRT-PCR	TBS on T, T + 2, and T + 3; qRT-PCR on T, T + 3, and select other days as a secondary method
DSM265	2016	Primary: qRT-PCR; secondary: TBS	qRT-PCR daily through end of treatment (T + 3) and every 1–3 d thereafter until negative; TBS through T + 3 as a secondary method
VTEU 11-0042/CVac	2017	qRT-PCR	Groups 1 and 2: qRT-PCR daily until 2 consecutive negative results; group 3: qRT-PCR daily until 1 negative result on or after T + 2
VTEU 14-0088/PfGAP3KO	2018–2019	qRT-PCR	qRT-PCR daily through T + 3, then at the discretion of investigators
USSPZV6^a^	2021–2022	qRT-PCR	qRT-PCR on T and at end of study

Abbreviations: qRT-PCR, quantitative reverse-transcription polymerase chain reaction; T, first day of treatment; T + 2, 2 days after T; T + 3, 3 days after T; TBS, thick blood smear.

a Recently completed study (S. Hoffman, personal communication).

It should be highlighted that the data presented here pertain to treatment with FDA-approved antimalarial drugs; studies of experimental antimalarial drugs still require intensive testing and follow-up after experimental drug administration. In addition, even in the context of use of FDA-approved treatments, CHMI study participants should still be carefully monitored for malaria-related symptoms and be able to receive on-demand testing when needed. Nonetheless, the data presented here suggest that qRT-PCR testing for the 18S rRNA biomarker on a reduced schedule has been, and can continue to be, safely used in place of daily TBSs to monitor and confirm infection clearance in CHMI trial participants after administration of FDA-approved treatment.

## Supplementary Material

ofad202_Supplementary_Data

## Supplementary Data

Supplementary materials are available at *Open Forum Infectious Diseases* online. Consisting of data provided by the authors to benefit the reader, the posted materials are not copyedited and are the sole responsibility of the authors, so questions or comments should be addressed to the corresponding author.
