# Supplementary material for: Plasmodium 18S Ribosomal RNA Biomarker Clearance After Food and Drug Administration–Approved Antimalarial Treatment in Controlled Human Malaria Infection Trials
Source: Open Forum Infect Dis. 2023 Apr 13;10(5):ofad202. doi: 10.1093/ofid/ofad202 (PMC10230565; doi:10.1093/ofid/ofad202)
Supplement: ofad202_Supplementary_Data [file ofad202_supplementary_data.docx]

Supplemental Figure 1. Kaplan-Meier plots showing the probability of being positive by TBS and qRT-PCR by study. “X” denotes one or more participants whose data were censored at the indicated time point.

**
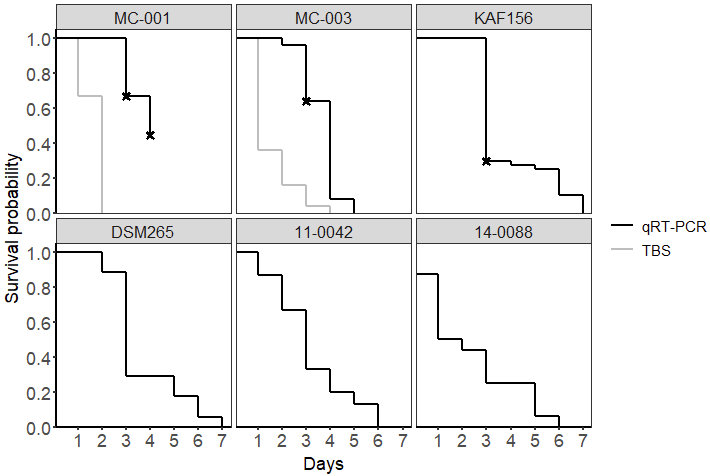
**
